# DNA segregation under Par protein control

**DOI:** 10.1371/journal.pone.0218520

**Published:** 2019-07-18

**Authors:** Lavisha Jindal, Eldon Emberly

**Affiliations:** Physics Department, Simon Fraser University, Burnaby, British Columbia, V5A 1S6, Canada; University of Oklahoma, UNITED STATES

## Abstract

The spatial organization of DNA is mediated by the Par protein system in some bacteria. ParB binds specifically to the *parS* sequence on DNA and orchestrates its motion by interacting with ParA bound to the nucleoid. In the case of plasmids, a single ParB bound plasmid is observed to execute oscillations between cell poles while multiple plasmids eventually settle at equal distances from each other along the cell’s length. While the potential mechanism underlying the ParA-ParB interaction has been discussed, it remains unclear whether ParB-complex oscillations are stable limit cycles or merely decaying transients to a fixed point. How are dynamics affected by substrate length and the number of complexes? We present a deterministic model for ParA-ParB driven DNA segregation where the transition between stable arrangements and oscillatory behaviour depends only on five parameters: ParB-complex number, substrate length, ParA concentration, ParA hydrolysis rate and the ratio of the lengthscale over which the ParB complex stimulates ParA hydrolysis to the lengthscale over which ParA interacts with the ParB complex. When the system is buffered and the ParA rebinding rate is constant we find that ParB-complex dynamics is independent of substrate length and complex number above a minimum system size. Conversely, when ParA resources are limited, we find that changing substrate length and increasing complex number leads to counteracting mechanisms that can both generate or subdue oscillatory dynamics. We argue that cells may be poised near a critical level of ParA so that they can transition from oscillatory to fixed point dynamics as the cell cycle progresses so that they can both measure their size and faithfully partition their genetic material. Lastly, we show that by modifying the availability of ParA or depletion zone size, we can capture some of the observed differences in ParB-complex positioning between replicating chromosomes in *B. subtilis* cells and low-copy plasmids in *E. coli* cells.

## Introduction

Bacteria are able to generate a wide variety of dynamic spatial patterns on the sub-micron scale even though many of their molecular parts rapidly mix due to diffusion. Within the cell, patterns range from periodic oscillations [1, 2] to the stable positioning of proteins asymmetrically at the cell poles [3, 4] (for a review see [5–7]). These spatial patterns serve many essential functions from cell division to signal reception [5, 8]. One particular protein system that is required for the faithful distribution of genetic material between daughter cells is the Par system. In *Caulobacter crescentus*, ParB binds at the origin of the newly replicated chromosome and moves uni-directionally up a gradient of ParA protein towards the opposite cell pole [4, 9]. In *Escherichia coli* it positions low-copy plasmids equi-distantly along the cell prior to cell-division so that both daughter cells inherit equal numbers [10]. A recent study suggests that the ParABS system, in conjunction with other complexes, may also be the driving mechanism behind the chromosome oscillations observed in *Bacillus subtilis* [11].

The complex dynamics of the Par system results from the specific interactions between the two proteins, ParA and ParB. Assays have revealed that ParA, an ATPase, forms dimers in the presence of ATP that are then competent to bind non-specifically to the bacterial nucleoid [12]. ParB molecules bind cooperatively to the DNA upstream of the *cis*-acting *parS* site to form a high-order nucleoprotein: the ParB-*parS* complex [13, 14]. Although the specific details of the interaction are not confirmed, this DNA bound ‘partition complex’ interacts with the nucleoid bound ParA and serves to stimulate the auto-hydrolysis of the attached ATP, causing ParA-ADP to dissociate from the nucleoid [15, 16]. The liberated ParA-ADP molecules diffuse within the cytoplasm, are rephosphorylated and subsequently rebind the nucleoid [17]. The result of these interactions is that the partition complex moves towards regions of higher bound ParA-ATP, leaving a wake of depleted ParA behind it. This wake is eventually rebound by ParA as it is reconverted to ParA-ATP.

Live imaging of ParB bound low copy plasmids *in vivo* interacting with the ParA bound nucleoid reveal complex spatial dynamics generated by this two protein system [18]. In cells that possess a single plasmid, it is observed to move from pole to pole, out of phase with ParA protein concentration [19, 20]. However, more often than not, these single plasmids replicate on their trajectory leading to altered dynamics due to the additional plasmid. With two or more ParB bound plasmids in a cell, the plasmids are seen to perform quasi-oscillations, attracting and repelling each other through the dynamic pattern of ParA on the nucleoid. In cells whose division has been inhibited, the result of the Par dynamics is to position the replicating plasmids equi-distantly along the cell length [20]. Nucleoid lengths reported in [18] show that more than half of the cells of a population of *E. coli* have nucleoid lengths significantly longer or shorter than the standard length (∼ 2*μ*m) and yet have robust genetic segregation. Given the observations, it is unclear whether the plasmids are undergoing stable oscillations or settling towards stable fixed point positions along the cell. Due to the constant appearance of new plasmids as the cell grows, untangling the true dynamics from the noise is challenging.

The Par system is also involved in chromosomal segregation within other bacteria (*C. crescentus*, *B. subtilis*, *P. aeruginosa*; *E. coli* chromosomes do not encode the ParABS system) and recent work has highlighted the differences between the dynamics of two F-plasmid foci in *E. coli* and two segregating chromosome origins within *B. subtilis* in cells with equal nucleoid length [18]. While the pair of F-plasmid foci segregate and settle near quarter-length positions, the *B. subtilis* chromosome origins were found closer to the nucleoid poles during the process of separation. Additionally, the plasmid foci are centred axially while the chromosome origins are offset in the radial direction. Although, the same ParABS system has been found to assist segregation processes in both these systems, the extent to which the segregation machinery is controlled by this system and its effect on the dynamics of partition complexes is not yet well understood.

Various molecular models have emerged to explain the mechanical and chemical aspects of this two-protein system that causes directional motion in a fluctuating thermal environment. One set of models proposes that the ParA-ATP forms polymers, similar to actin filaments, when associating with the nucleoid and a depolymerization force pulls the ParB bound partition complexes directionally [10, 19]. In other models, ParA-ATP dimers bind the nucleoid uniformly and the ParA hydrolysis stimulating activity of the ParB leads to the spontaneous creation of a ParA-ATP gradient around the partition complex [21, 22]. The ParB complex climbs this ParA-ATP gradient to execute directional motion. Translocation force is thought to be generated due to the elastic properties of the underlying nucleoid and it is transmitted to the ParB complex through transient bonds with the nucleoid bound ParA [23]. These ParA-ParB bonds further assist unidirectional motion by tethering the ParB complex which quenches its lateral diffusion [22]. Stochastic simulations of the above models show that oscillations can be generated but it can be challenging to determine whether the dynamics represents a limit cycle or stochastic motion around a stable fixed point, especially for the case where there are multiple plasmids.

Here we present a deterministic model that complements the prior modelling efforts [19, 22, 24–26] and can address the unanswered questions about the nature of the dynamics. This study extends prior work [21] that was developed to model the landmark *in vitro* experiment where micron sized ParB bound beads were found to move ballistically along a ParA bound substrate [27]. It is based on random binding of ParA-ATP to a finite sized substrate (such as the nucleoid) and assumes that the net force on each ParB bound complex is due to the elastic restoring forces generated by the interaction between DNA bound ParB and the ParA bound nucleoid. The dynamics of the model depends on the following parameters: the substrate (e.g. nucleoid) length, number of ParB complexes, the total amount of ParA, the hydrolysis rate and a dimensionless factor set by the ratio of the range of the force to that of the ParA depletion zone. We consider two possible mechanisms for ParA rebinding: a well buffered environment and a constant ParA rebinding rate (resembling the *in vitro* experiment) or a dynamic rate of rebinding where the amount of ParA available is limited (resembling bacterial environments *in vivo*). Within finite systems with a constant rate of ParA rebinding, partition complexes either oscillate or settle into fixed positions along the length of the substrate based on total ParA and the hydrolysis rate of substrate bound ParA. We find that above a minimum substrate size, partition complex dynamics are independent of partition complex population and system size. Conversely, for the case where ParA is limited and rebinding rates fluctuate in time, the dependence of ParB-bound partition complex motion on substrate length and number of complexes is complex and results in counteracting mechanisms. We find that as more ParB-complexes are added to the cell, more substrate bound ParA is liberated. This can push a stable arrangement of ParB-complexes into oscillations. Counteracting this is the growing substrate, which serves to dilute the added ParA and return the system to stable fixed points. Such competing mechanisms could prove useful to a cell when segregating plasmids as it uses oscillations to explore the growing space, but eventually settles into a stable plasmid arrangement so that the genetic material can be partitioned faithfully.

In the final section of our results, we extend our model to two dimensions with limited ParA resources. The key dependencies of partition complex dynamics on system parameters remain the same and a variety of spatial patterns is generated through the combination of oscillatory motion along one axis with stable organization along the other. We compare the ParA and ParB spatial patterns of two partition complexes on a rectangular substrate to those observed for a pair of F-plasmids in *Escherichia coli* and chromosomal origins in *Bacillus subtilis* [18] and identify the system parameters that generate the observed distinctive spatial patterns on identical substrate sizes. Our results show the versatility of the Par system in orchestrating various spatial patterns in different bacterial cells despite having only two proteins linked through a simple Brownian ratchet biochemistry.

## Model

In previous work [21] we presented a deterministic model for the experimentally observed motion of ParB coated micro-beads on a substrate that is uniformly bound by ParA-ATP dimers [27]. Our model was able to replicate the persistent unidirectional motion of the beads. Here we extend that model and confine it to finite substrates such as the volume of the nucleoid within a cell. Previously, we only considered a constant rate of ParA rebinding (as found *in vitro* where a buffer supplies constant amounts of ParA for rebinding). Here we also consider the case of a variable rebinding rate that arises when there are limiting amounts of ParA, as might be the case *in vivo*.

**Fig 1:**
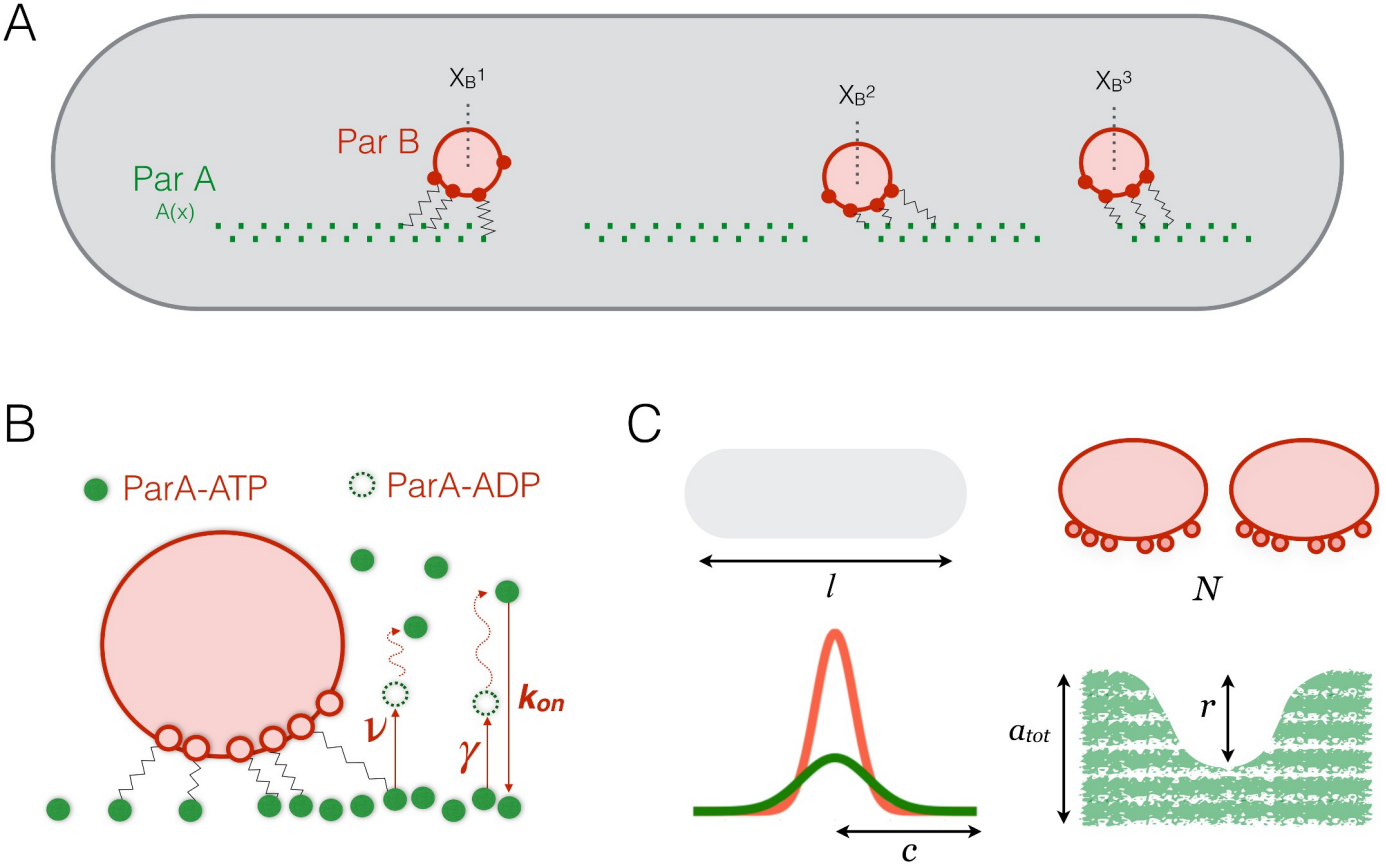
Model schematic and system parameters. (A) A finite one-dimensional substrate is bound by a ParA-dimer concentration, given by *A*(*x*) (green). It attracts multiple ParB bound partition complexes (red) with centre of mass positions given by $X_{B}^{i}$. These partition complex foci avoid each other due to their attraction for ParA dimers which are reduced in the regions surrounding a partition complex. (B) The random microscopic forces applied on each complex due to elastic ParA-ParB bonds are harnessed into a forward force. ParA is bound to the substrate in its ParA-ATP state and is released from the substrate when it is hydrolysed by the ParB into ParA-ADP at a rate *ν*. This hydrolysis also occurs at a smaller, non-stimulated rate given by *γ*. This ParA-ADP phosphorylates into ParA-ATP and rebinds the substrate at a rate *k*_*on*_ after a time-delay. (C) Schematic of the five parameters that affect complex dynamics in our dimensionless model to give the full range of observed ParB-complex behaviour *in vivo*. These are: the length of the substrate (*l*), the number of partition complex foci on the same substrate (*N*), the parameter *c* which gives the ratio of the length scale at which ParA is hydrolysed to the length scale at which ParB-complex foci experience a force of attraction from the substrate bound ParA, the total initial ParA in the system (*a*_tot_) and the hydrolysis factor, *r*, which gives the ratio of ParB-stimulated ParA hydrolysis rate (*ν*) to non-stimulated ParA hydrolysis rate (*γ*).

In Fig 1(a) we show a schematic of our model for the ParA-ParB system. Multiple ParB bound complexes can move within a finite volume that is bound by ParA-ATP dimers, such as the nucleoid. The ParA protein primarily exists in two interconvertible forms within the system: substrate bound ParA-ATP that attracts ParB and cytoplasmic ParA-ADP which is well mixed and rebinds the substrate as it phosphorylates to ParA-ATP. Given the cylindrical shape of most bacterial cells, and that ParB-complex motion has been approximated to be unidirectional, we consider the long axis to be our *X* direction along which the primary motion occurs (we extend to motion along the radial axis, *Y*-direction, in the last section). We track the dynamics of two quantities in our simulations: the position of the centre of mass of each partition complex ($X_{B}^{i}\left(t\right)$) and the time dependent concentration of bound ParA-ATP dimers along the *X*-axis of the substrate, *A*(*X*, *t*). Based on the recent study [24], we assume that ParB-complex motion arises due to the DNA relay-mechanism that is harnessed by the ParB complex through transient bonds with the DNA associated ParA-ATP dimers. ParA bound to the nucleoid, fluctuates in position and interacting ParB-complexes can capture these fluctuations leading to a cumulative elastic restoring force on the complex. These ParA loci have been observed to fluctuate within a harmonic potential over a length range of *σ*_*F*_ (≈ 100 nm) which gives a corresponding effective elastic constant for their restoration ($k_{X}=k_{B}T/\sigma_{F}^{2}$). Our key assumption is that when a partition complex with its centre of mass at $X_{B}^{i}$ comes in contact with ParA-ATP at a position *X*, the elastic cost of deforming the system is given by $E=\frac{1}{2}\frac{k_{B}T}{\sigma_{F}^{2}}{\left(X-X_{B}^{i}\right)}^{2}$. We assume that ParA-ParB complex formation and dissociation comes to a steady state so that the amount of force exerted on the complex is proportional to the probability of deforming the system, *e*^−*E*/*k*_B_*T*^, and the concentration of ParA at *X*, *A*(*X*). Each ParA-ParB contact then generates an elastic force on the centre of mass of the complex equal to $\frac{k_{B}T}{\sigma_{F}^{2}}\left(X-X_{B}^{i}\right)$. Finally, the total force on a complex at a given time can be calculated by integrating all these forces over the entire substrate. Assuming that the ParB-complexes are in the overdamped regime, the resulting equation of motion for the centre of mass of the *i*^*th*^ complex is given by:

$$\begin{array}{c} \xi \frac{dX_{B}^{i}}{dt}=Q\int_{-L/2}^{L/2}dX\;\frac{k_{B}T}{\sigma_{F}^{2}}e^{-\frac{{\left(X-X_{B}^{i}\right)}^{2}}{2\sigma_{F}^{2}}}\left(X-X_{B}^{i}\right)A\left(X,t\right), \end{array}$$ (1)

where *ξ* is the drag coefficient of the ParB bound partition complex, *L* is the length of the substrate (nucleoid), and *Q* is a geometric factor that accounts for the reduction of a three dimensional space into a one dimensional system (see supporting information for the calculation of this factor).

ParA protein exists in the cell in two principal forms, the substrate bound ParA-ATP form and the freely diffusing cytoplasmic form which is predominantly composed of ParA-ADP. The substrate bound ParA-ATP is released from the nucleoid when it undergoes hydrolysis, which is stimulated by the ParB-complex apart from occurring at a natural rate. The released ParA-ADP undergoes phosphorylation in the cytoplasm and rebinds the substrate as ParA-ATP. Importantly, the directionality of partition complex motion is maintained by this hydrolysis cycle of the bound ParA-ATP which is removed in the wake of the partition complex, biasing its motion in the forward direction. In Fig 1(b) we highlight the chemical reactions that govern ParA-ATP hydrolysis and substrate binding in our model. The system is initialized with a finite concentration of ParA, *A*_tot_, which is divided into substrate-bound ParA-ATP, *A*(*X*), and a freely diffusing portion within the system buffer (or cytoplasmic ParA-ADP within the cell). The natural rate of ParA-ATP hydrolysis is given by *γ* and the ParB stimulated rate is given by *ν*. In our model, we assume that the ParB-complex stimulated hydrolysis of the bound ParA-ATP is modulated by a Gaussian profile. This profile accounts for the likelihood of the ParB-plasmid complex interacting with ParA at a given separation, liberating a greater concentration of ParA-ATP closer to its centre of mass and thus, depends on the distance of ParA separation. To keep things general and account for intermediate states in the ParA-ParB chemistry we make a mathematical simplification and model the length scale of the ParA hydrolysis (by the ParB complex) as a multiple of the length scale of ParA fluctuations that mediate the force. Hence, *cσ*_*F*_ gives the effective length scale over which the ParB mediates the hydrolysis of ParA where *c* is a dimensionless factor. Low values of *c* lead to smaller ParA depletion zones around the partition complex while higher values create wider depletion zones (the magnitude of ParA hydrolysis is governed by *ν*). This finally gives the effective distance dependent probability for ParA to unbind the substrate due to ParB stimulation as $\text{exp}(\frac{1}{2}\frac{k_{B}T}{\sigma_{F}^{2}c^{2}}{\left(X-X_{B}^{i}\right)}^{2})$. The rebinding concentration of cytoplasmic ParA-ATP to the substrate is governed by a rate, *k*_*on*_, multiplied with the available cytoplasmic ParA concentration, *A*_tot_ − 〈*A*(*X*)〉. On the timescale of partition complex motion, we take the ParA-ADP to ParA-ATP turnover time to be quick and consider the effective ParA available for rebinding to be equal to the concentration difference between total ParA and bound ParA. Combining these reactions, we arrive at our equation for the dynamics of the ParA-ATP substrate bound concentration,

$$\begin{array}{c} \frac{\partial A(X,t)}{\partial t}=k_{on}\left(A_{\text{tot}}-\left\langle A\left(X,t\right)\right\rangle \right)-\gamma A\left(X,t\right)-\nu \sum_{i=1}^{N}\exp\left[-\frac{{\left(X-X_{B}^{i}\right)}^{2}}{2\sigma_{F}^{2}c^{2}}\right]A\left(X,t\right), \end{array}$$ (2)

where $\langle A\left(X,t\right)\rangle =\frac{1}{L}\int_{-L/2}^{L/2}dXA\left(X,t\right)$ is the average amount of bound ParA concentration and *L* is the length of the substrate.

We can dedimensionalize the above equations by rescaling position, time and concentrations and we shall use lowercase variables to represent dimensionless quantities (for more details see the supporting information). All spatial dimensions are scaled by the characteristic length scale *σ*_*F*_, time is rescaled to the dimensionless quantity, *τ* (= *νt*), and concentrations are rescaled by a characteristic concentration *A*_0_ = *ξνσ*_*F*_/*πk*_B_*T*. We also use the Stoke Einstein relation to replace the drag on the partition complex, *Dξ* = *k*_B_*T*. We can rescale all the concentrations; for example *A*(*x*) = *A*_0_*a*(*x*). Based on previous experimental and modelling work, we further simplify the system by assuming the on rate for rebinding is roughly the same as the ParB stimulated off rate, *k*_*on*_ ≈ *ν* [17]. This leads to the following dimensionless equations:

$$\begin{array}{c} \frac{dx_{B}^{i}}{d\tau }=\int_{-l/2}^{l/2}dx\;e^{-\frac{{\left(x-x_{B}^{i}\right)}^{2}}{2}}\left(x-x_{B}^{i}\right)a\left(x,\tau \right), \end{array}$$ (3)

and

$$\begin{array}{c} \frac{\partial a(x,\tau )}{\partial d\tau }=\left(a_{\text{tot}}-\left\langle a\left(x,\tau \right)\right\rangle \right)-\frac{a(x,\tau )}{r}-\sum_{i=1}^{N}\exp\left[-\frac{{\left(x-x_{B}^{i}\right)}^{2}}{2c^{2}}\right]a\left(x,\tau \right), \end{array}$$ (4)

where *r* = *ν*/*γ* is the hydrolysis factor that gives the ratio of the rate of stimulated ParA hydrolysis, *ν* to the rate of non-stimulated ParA hydrolysis, *γ*. To get a sense for the parameter values that one might find *in vivo*, we use reported values of *ν* ≈ 0.1/s [28], *σ*_*F*_ ≈ 100 nm [24], and use a plasmid diffusion constant of *D* = 0.0003*μ*m^2^/s [25] to find *A*_0_ = 1760 nM. In the results that follow, we discuss system dynamics in terms of our dimensionless model parameters, converting into dimensionful quantities as required.

Our dedimensionalized system has only five parameters which are schematically depicted in Fig 1(c). They are, the number of partition complexes (*N*), the length of the substrate (*l*), the total ParA concentration (*a*_tot_), the ParB stimulated ParA hydrolysis factor (*r*), and the lengthscale ratio *c*. Depending on parameter values the dynamics can either decay to stable fixed points or oscillate. Oscillations can either be spatially confined to well defined domains or intermingled where complexes can invade each others territories. We now map out the phase space of our model and show how limiting ParA resources leads to interesting competing effects as both complex number and substrate length are changed.

## Results

### Partition complex dynamics with constant ParA rebinding rate

In this section we provide an exhaustive mapping of the dynamics of single and multiple partition complexes on finite substrates where the rate of ParA rebinding is constant and only set by the total amount of ParA, *a*_tot_. In particular, for a fixed set of parameter values we will determine whether the complexes decay to stable positions along the substrate length or execute sustained oscillations. The constant rate of rebinding is found by assuming that the presence of the ParB complexes has no effect on the buffer levels of ParA (likely to be true *in vitro*) and so we solve Eq 4 at steady state for *a*(*x*) in the absence of complexes, leading to $a\left(x\right)=ra_{\text{tot}}/\left(1+r\right)$. Thus the rate of rebinding $\left(a_{\text{tot}}-a\left(x\right)\right)=a_{\text{tot}}/\left(1+r\right)$ which is independent of the number of plasmids and the substrate length.

**Fig 2:**
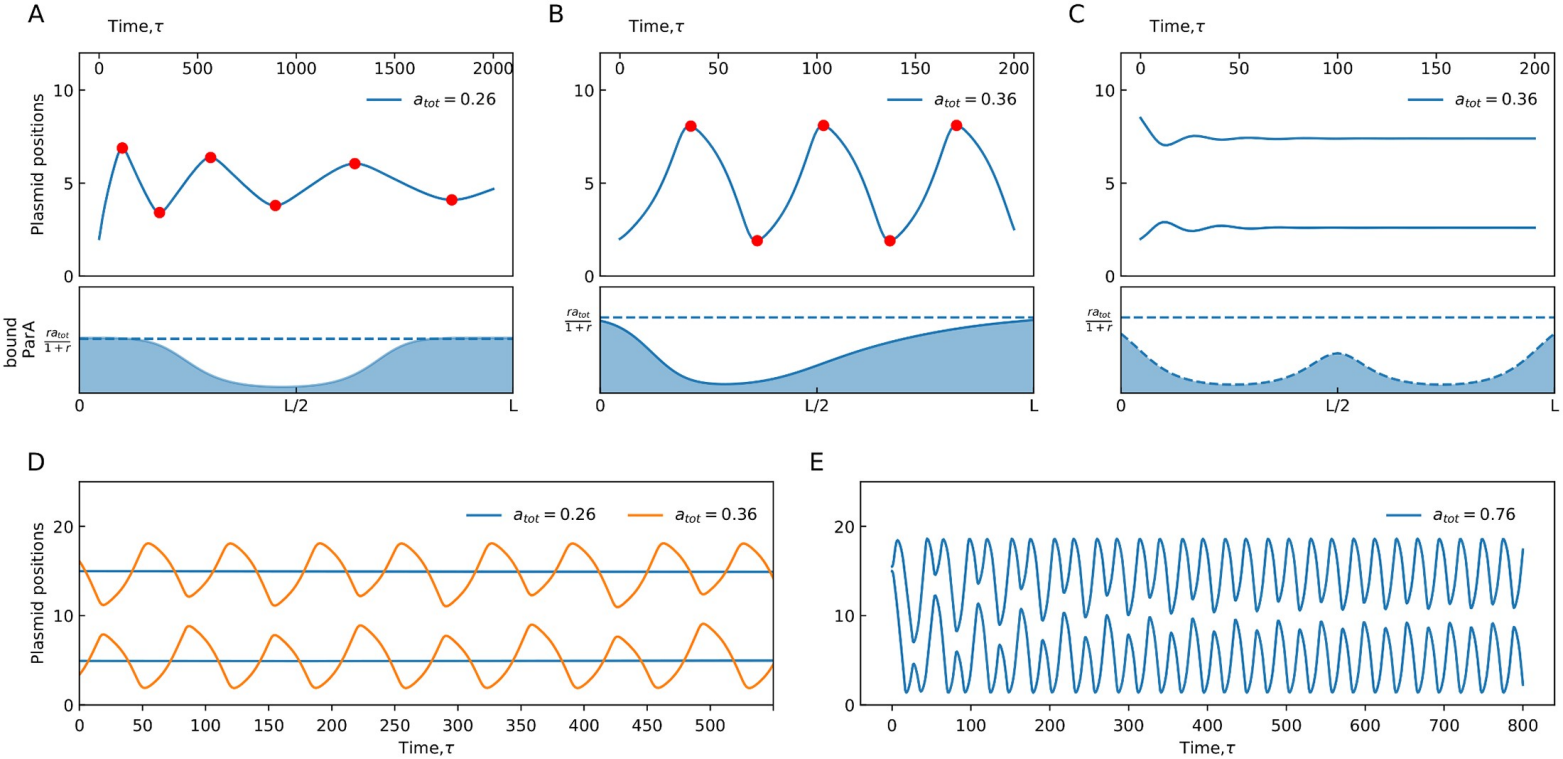
Partition complex foci trajectories on a finite substrate. (A) (Upper) Partition complex position as a function of time, *τ*, for a single copy on a substrate of length 10 (*a*_tot_ = 0.26, *r* = 8 and *c* = 1). (Lower) The instantaneous distribution of the bound ParA as a function of position along the nucleoid length, *a*(*x*, *τ* = 2000). (B) Same as (A) with *a*_tot_ = 0.36. Red circles mark the extremas of the oscillatory trajectories and are used to calculate the time period of oscillations. The lower panel shows the distribution of bound ParA, *a*(*x*, *τ* = 200). (C) Partition complex foci positions for two complexes on a substrate of length 10 (*a*_tot_ = 0.36, *r* = 8 and *c* = 1) along with the resultant instantaneous bound ParA profile, *a*(*x*, *τ* = 200). (D) Two partition complexes on a substrate of length 20 with a total ParA concentration of *a*_tot_ = 0.26 (blue) and *a*_tot_ = 0.36 (orange). All other parameter values are kept the same (*r* = 8, *c* = 1). (E) Partition complex foci positions for two copies on a substrate of *l* = 20 and *a*_tot_ = 0.76 form intermingled trajectories that are long lived transients which eventually segregate (*r* = 8, *c* = 1).

In Fig 2 we show some of the possible dynamics of the model along with the resultant ParA distribution profiles for some of these sample cases. Fig 2A shows a decaying trajectory to a fixed point for a single partition complex on a substrate of size *l* = 10 with a total ParA concentration of *a*_tot_ = 0.26. The distribution of the bound ParA as a function of position along the nucleoid shows how there is no ParA imbalance around the partition complex and that the mechanism of ParA removal by the ParB-complex is balanced by the ParA rebinding such that a stable ParA profile is formed around the complex. As the ParA concentration is increased to *a*_tot_ = 0.36 the single ParB-complex executes sustained oscillations (Fig 2B), indicating that greater ParA resources support oscillatory behaviour through the creation of a sustained ParA-ATP concentration gradient around the complex. We measure the amplitude as well as time period of oscillatory trajectories (see Methods for details) to determine if trajectories are sustained oscillations or decaying to stable spatial fixed points and use these to construct phase portraits presented below.

In Fig 2C we increase the number of partition complexes to two on the same substrate of *l* = 10 while keeping the total ParA concentration fixed at *a*_tot_ = 0.36 and it can be seen that oscillatory motion is subdued. The resultant ParA profile shows how the ParB-complexes maximize their interaction with the bound ParA by settling at quarter length positions along the nucleoid. This implies that spatial confinement, due to multiple partition complex foci, can subdue oscillatory motion even though sufficient ParA resources are available. To test this, we increase the length of the substrate to *l* = 20 in Fig 2D and vary ParA concentrations. While oscillatory behaviour is recovered for a total ParA concentration of *a*_tot_ = 0.36, it is not recovered for the lower value of *a*_tot_ = 0.26 (at which a single complex did not oscillate either). These explorations of length and ParA concentrations imply that there is a minimum ParA concentration below which oscillations cannot be triggered irrespective of substrate length. Above this minimum ParA concentration, the substrate space available determines whether ParB-complexes will oscillate or not. Two or more complexes can both effectively repel and move towards each other due to their coupled interaction with the ParA bound substrate. Complexes always move to regions of higher ParA concentration but do not invade another complex’s depletion zone. Thus, a pair of partition complexes settles into the quarter length positions when there are insufficient ParA resources or insufficient substrate space. Interestingly, as ParA concentration is increased further partition complexes can also create intermingled trajectories that have long lived transients that eventually separate into segregated oscillatory trajectories (Fig 2E). Evidence of such intermingling has been observed experimentally in [19] and obtained computationally in stochastic models [24] but the time period for such trajectories (obtained from a drastic increase in ParA in our deterministic model) is much smaller than the time periods associated with experimental plasmid oscillations (∼ 20mins) in *E. coli* cells.

**Fig 3:**
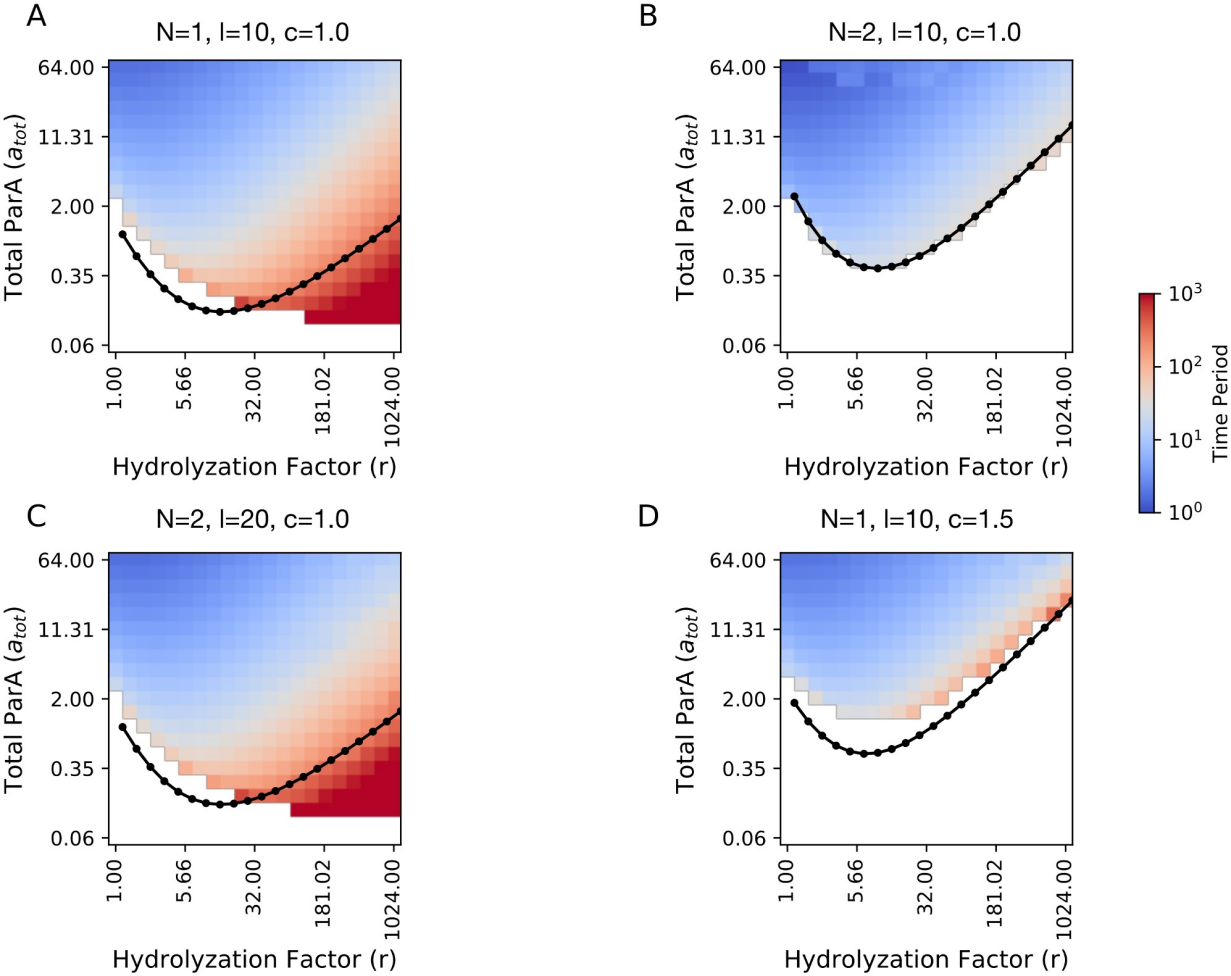
Phase portraits show regions of oscillatory and non-oscillatory behaviour *in vitro*. (A) Time period of oscillation for a single partition complex with *c* = 1 on a substrate of *l* = 10 as a function of total initial ParA, *a*_tot_, and hydrolysis factor, *r*. The white region constitutes the phase space over which the complex decays to a stable position. We obtain the boundary (black line) that separates regions of oscillatory motion from non-oscillatory motion from analytic calculations. (B) Time period of oscillation of a partition complex placed on a substrate (*l* = 10) along with another partition complex as a function of *a*_tot_ and *r*. Both partition complexes are identical and have *c* = 1. The analytic boundary is calculated by considering a single plasmid of *c* = 1 on a substrate of *l* = 5. (C) The average time period of oscillation of two identical partition complexes placed on a substrate (*l* = 20) as a function of *a*_tot_ and *r*. Both partition complexes have *c* = 1 and we recover the phase portrait of a single partition complex on a substrate of length 10. The analytic boundary is calculated by considering a single plasmid of *c* = 1 on a substrate of *l* = 10. (D) Same as (A) with *c* = 1.5.

In Fig 3A we present a phase portrait of the time period of the oscillations of a single partition complex as a function of total initial ParA, *a*_tot_ and the stimulated removal factor, *r*, for a substrate of dimensionless length, *l* = 10 and *c* = 1. The white region corresponds to infinite time period and stable fixed point dynamics, while oscillatory motion has time periods ranging from 10-10^3^ in *τ*. This phase diagram again shows that on a fixed length substrate there is a minimum amount of ParA needed to generate oscillations and its value depends on the rate of removal. Below this minimum ParA concentration, the partition complex assumes a central position along the length of the substrate. Above this minimum ParA concentration, oscillatory behaviour becomes more rapid as the ParA increases and less rapid as the stimulated rate of removal increases. At lower values of *r*, we find that higher concentrations of ParA are required before oscillations are possible. These results indicate that a partition complex requires a balance between ParA concentrations and ParB-stimulated ParA hydrolysis to orchestrate movement of a desired velocity across the substrate. Our results are also in agreement with the phase plots found from stochastic simulations of the *in vitro* ParA-ParB system in the range of high removal rates [22].

On simulating a single partition complex on substrates of greater length, keeping all other parameters the same, we observe that the parameter regimes permitting oscillations are unaffected by length. Although an increase in substrate size leads, not surprisingly, to an increase in the time period of an oscillating complex, the triggering of oscillatory behaviour at a particular combination of *a*_tot_ and *r* values remains unchanged (see S1 Fig). This is also not surprising since the rebinding rate of ParA is independent of system size, leaving the local ParA gradient around the complex unchanged. If the substrate size is reduced, however, the role of spatial confinement would become evident and the parameter regime permitting oscillations would diminish.

In Fig 3B, we show how the phase boundary between oscillations and stable fixed points changes when a second partition complex is added to the system. We find that a higher *a*_tot_ is required to trigger oscillations compared to the single plasmid case. This is because adding partition complexes on the same substrate leads to spatial confinement which hinders oscillatory motion. On increasing the length of the substrate to *l* = 20, and reducing the effects of confinement for the two-complex system, we recover a phase plot that is identical to the single complex on *l* = 10 case (Fig 3C). We will see that this tug-of-war between confinement that limits oscillations and substrate growth that revives them, leads to even more nuanced dynamics when ParA amounts are limited.

Another parameter that governs ParB complex motility is the factor *c* which is the ratio of the spatial range of ParB stimulated ParA removal to the range of the force from the DNA-substrate. As previously discussed, we include this factor to keep our model general and account for variations in the rates associated with the underlying ParA-ParB chemistry. Variations in the rates of ParA to ParA-B conversion and ParA-B complex to cytosolic ParA could lead to differences in the spatial distance over which the partition complex feels a translocation force and the distance over which ParA-ATP hydrolysis occurs. These variations could possibly arise from differences in the ParB population on the partition complex or structural differences in the partition complex which are not yet known. In Fig 3D we show the effect of this system parameter on the phase plot by increasing this factor to *c* = 1.5 from *c* = 1.0. This increase, which creates a larger depletion zone around the complex, causes oscillations to commence at higher values of *a*_tot_ compared to the results of Fig 3A. This observed decrease in the phase space of oscillatory behaviour at higher *c* is similar to increasing the spatial confinement of a complex that has a smaller value of *c* (compare Fig 3C and left region of Fig 3A).

Given that our model is deterministic, we are able to carry out linear stability analysis on the equations for the single ParB-complex system (see supporting information) and predict the boundary between the non-oscillatory and oscillatory solutions as a function of parameters (*c*, *r*, *a*_tot_, and *l*). The analysis is based on approximating the ParA dynamics with the motion of the first moment of the ParA distribution. Intuitively, we take this to be the position of the minimum of the wake of the ParA distribution, that we label *x*_*A*_, which lags the position of the ParB complex, *x*_*B*_. We take as the boundary to be those parameter values that make the perturbations to *x*_*A*_ and *x*_*B*_ decaying functions of time (i.e. a negative real part for all eigenvalues of the Jacobian matrix). Hence, for a given set of parameter values, *r*, *c*, and *a*_tot_, it can now be determined if slight perturbations grow and potentially lead to persistent oscillations or if these perturbations decay and the complex retain its stability. We calculate this boundary for a substrate of *l* = 10 and *c* = 1 for a single complex (overlaid black line in Fig 3A). Our linear approximation does poorly for large values of *r*, where long range interactions between the partition complex and substrate bound ParA lead to oscillations with extremely large time periods (∼ 10^3^). The analytic phase boundary calculations for a single complex can be further used to predict the phase boundaries of multiple complexes on substrates of differing lengths. Because of their mutual avoidance, two partition complexes on a substrate of *l* = 10 behave identically after the transients fade (Fig 2C–2E). Either complex on this substrate is similar in dynamical behaviour to a single complex on a substrate of *l* = 5. We find a good agreement between simulations and the analytical boundary calculated based on this argument in Fig 3B for two complexes with *c* = 1 on *l* = 10. Through repeating this comparison between simulations and analytic boundaries for greater number of complexes on differing substrate sizes, we notice that our analytic approximation is stronger when partition complexes are under stronger spatial confinement. Good agreement is also observed when we increase *c* to 1.5 (Fig 3D), the effect of which is similar to increasing confinement.

### Partition complex dynamics with limited ParA resources

We now consider the case of limited ParA resources, such that the rate of rebinding is variable as may be the case *in vivo*. Here the system is again initialized with a total ParA concentration, *a*_tot_, however, the cytoplasmic ParA that is available for rebinding is calculated as the difference between total ParA and average substrate bound ParA at a given time. Assuming fast diffusion and phosphorylation of cytoplasmic ParA, the ParA available for rebinding is given (as in Eq 4) by *a*_tot_ − 〈*a*(*x*, *τ*)〉, where 〈*a*(*x*, *τ*)〉 = ∫*a*(*x*, *τ*)/*l*. Thus the rebinding rate is dynamic, and bears closer resemblance to the environment *in vivo* where substrate size is small and cytoplasmic ParA concentrations are sensitive to complex number and changes to the substrate length.

**Fig 4:**
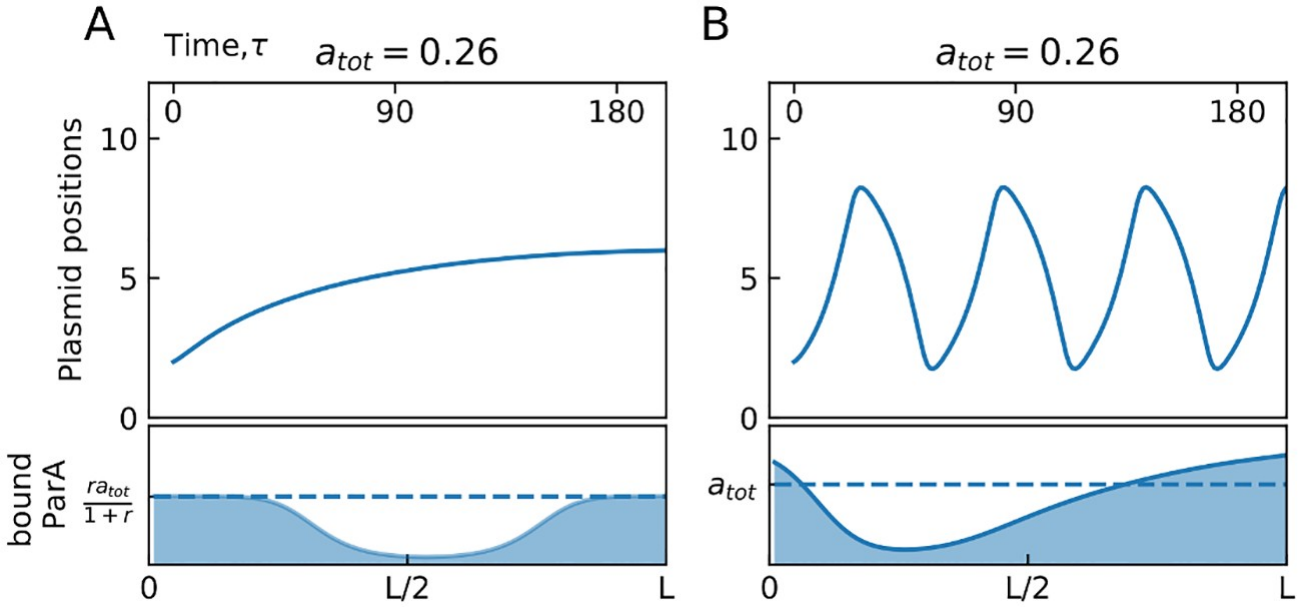
ParB-complex dynamics for different cases of ParA rebinding. (A) (Upper) Position as a function of time for a single partition complex on a substrate of length 10 as the rate of ParA rebinding is held constant (*a*_tot_ = 0.26, *c* = 1, *r* = 8). (Lower) The instantaneous distribution of ParA concentration as a function of position along the length of the nucleoid, *a*(*x*, *τ* = 200). (B) (Upper) Position as a function of time for a single partition complex on a substrate of length 10 as the total concentration of ParA in the system is held constant but the rate of ParA rebinding per site is variable (*a*_tot_ = 0.26, *c* = 1, *r* = 8). (Lower) The instantaneous distribution of ParA concentration as a function of position along the length of the nucleoid, *a*(*x*, *τ* = 200), for the case of variable rate of ParA rebinding.

In Fig 4 we show the differences in a ParB trajectory between having constant ParA rebinding or time dependent rebinding due to limited ParA. For the case with constant rebinding (Fig 4A), the maximum possible ParA concentration at a site is bounded at *ra*_tot_/(1 + *r*), determined by the balancing of the removal process by the rebinding process. For this case when the buffer supplies a constant ParA concentration of *a*_tot_ = 0.26 to a single partition complex it decays to the centre of the substrate. However, when the only constraint on the system is that the total ParA remain constant (such that the rate of rebinding at each site can vary with time) the ParA concentration that can bind a site is now unbounded as long as ParA is available in the cytoplasm (Fig 4A). Thus, oscillatory behaviour can now occur at *a*_tot_ = 0.26, where it did not when the system was buffered and had constant rebinding. This occurs because the ability of ParB-complexes to liberate ParA resources and add to cytoplasmic ParA available for rebinding is now accounted for in this case. Increased cytoplasmic ParA makes oscillations easier by rebinding to the substrate ahead of the complex and increasing the magnitude of the leading edge of the ParA profile.

**Fig 5:**
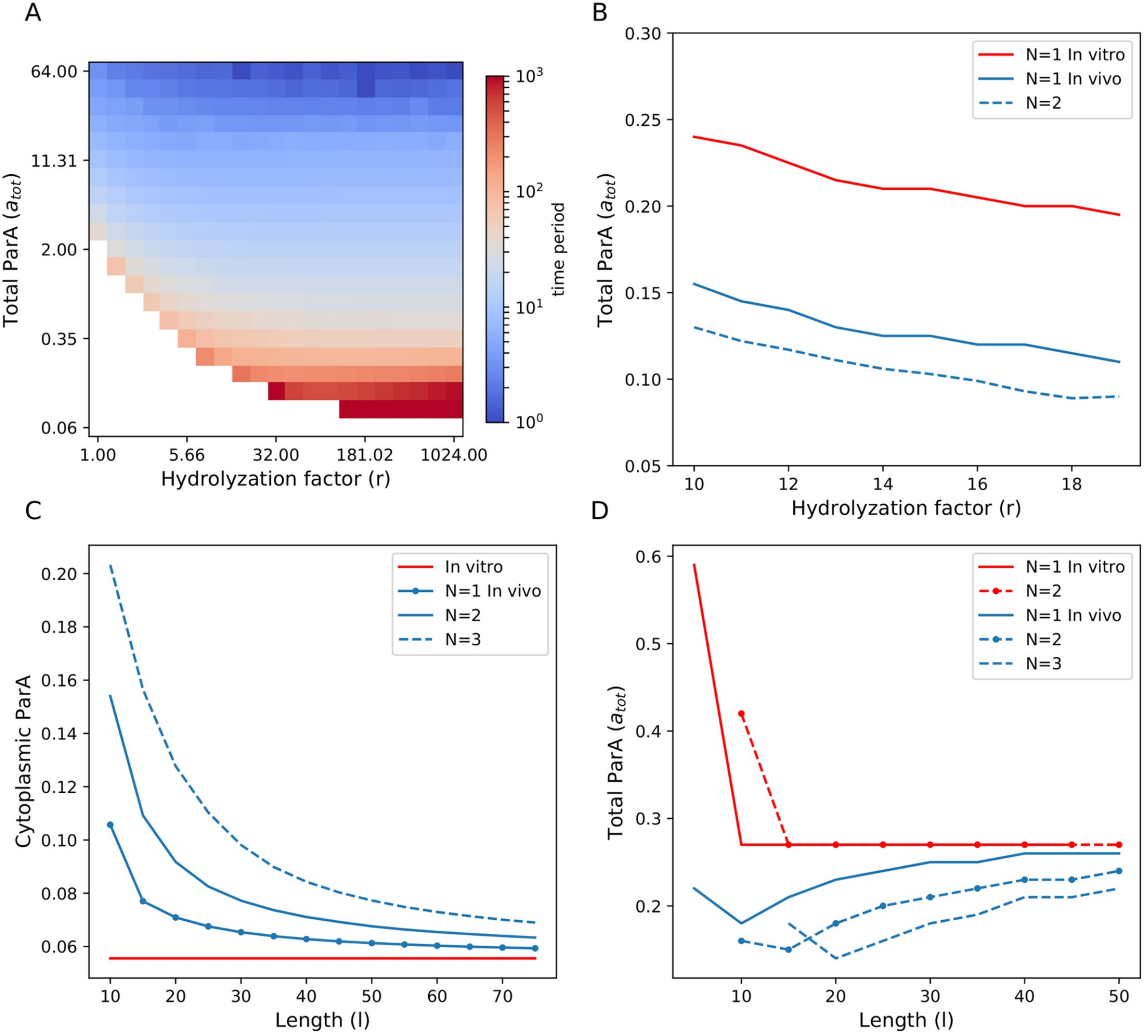
Par protein dynamics when ParA resources are limited. (A) Phase plot for a system with limited ParA resources and *l* = 20 (corresponding to a nucleoid size of 2*μ*m), showing the time period of single ParB-complex oscillations as a function of *r* and total initial ParA, *a*_tot_, with *c* = 1. The time period of oscillations ranges from 1000 to 10 (and smaller) over this chosen parameter regime. (B) The phase boundaries separating regions of oscillatory motion from regions of stable positions over a range of hydrolysis factors from 10 to 20 for an *in vitro* (red) system with a single complex and for *in vivo* (blue) systems with a single and a pair of complexes. (C) Cytoplasmic ParA available for binding for comparable *in vitro* (red line) and *in vivo* systems (blue lines) initiated with the same ParA concentration (*a*_tot_ = 0.5) and ParA hydrolysis factor (*r* = 8.0, *c* = 1.0) as a function of system size. (D) Minimum *a*_tot_ required to initiate oscillatory motion as a function of system size *in vitro*(red lines) and *in vivo*(blue lines) for multiple complexes at a fixed hydrolysis factor, *r* = 8, and *c* = 1.

In Fig 5A we show the phase portrait of the dynamics of a single partition complex when ParA resources are limited on a substrate of length, *l* = 20. Now, an increase in partition complex number can potentially aid oscillations by liberating more ParA to the cytoplasm. In Fig 5B, the *a*_tot_ boundary between oscillatory and decaying behaviour is shown as a function of removal rate as complex number is increased. The red curve shows the boundary for the constant rebinding case, which for *l* = 20 is the same for *N* = 1 or 2. However when ParA is limited, the boundary continues to shift to lower *a*_tot_ as the number of partition complexes is increased from one to two. Again this is due to increased liberation of ParA into the cytoplasm, which lowers the minimum amount of initial ParA required to initiate partition complex oscillatory motion.

In Fig 5C we show how the concentration of free ParA varies (keeping *a*_tot_ fixed) as the substrate length is changed. At large *l* limit, the system becomes insensitive to the number of ParB-complexes and tends to the constant rebinding case. When substrate size is small, the amount of free ParA increases and goes up with the number of complexes, making the onset of oscillations potentially easier for a larger number of partition complexes. This can lead to interesting dynamics when one considers the cell cycle of bacteria, which includes both a growing nucleoid and increasing plasmid copies. The growing nucleoid (substrate) dilutes out the free ParA, potentially leading to stable fixed points, whereas additional plasmids (ParB-complexes) potentially favour oscillations.

To bring all these ideas together, in Fig 5D we plot the required value of *a*_tot_ to generate oscillations as a function of substrate length (at a fixed removal rate) for both the constant rebinding case and variable rebinding as we vary the number of complexes. For the constant rebinding simulations (red lines) of a single and double partition complex system, we see that the minimum amount of ParA required to initiate oscillations remains the same as substrate length is increased, implying that the local ParA gradient around a complex is not altered by the total number of partition complexes on the substrate or the substrate’s length (as previously seen). At shorter lengths, however, confinement plays a role and a higher amount of *a*_tot_ is required to trigger oscillations in the two complex system compared to the system with a single complex. For limited ParA concentration (blue lines) such that rebinding rate is dynamic, the system is sensitive to both complex number and substrate length in complicated ways. In the limit of large length, all the systems (*N* = 1, 2, 3) can be seen to tend towards the amount of *a*_tot_ required to trigger oscillations in the constant rebinding rate case. In this regime, less ParA is required to trigger oscillations as complex numbers increase. At shorter lengths, however, this direct dependence of additional complexes adding more cytoplasmic ParA leading to the triggering of oscillatory motion at lower *a*_tot_ values is hampered as confinement effects also affect the system. For example, at *l* = 15, a three complex system requires higher ParA to trigger oscillations than a two complex system. Here, even though there is additional ParA in the cytoplasm due to increased complex number, there are also confinement effects at play because the substrate length is short. We show how this can lead to intriguing dynamics as a cell both grows and adds plasmids to its volume.

**Fig 6:**
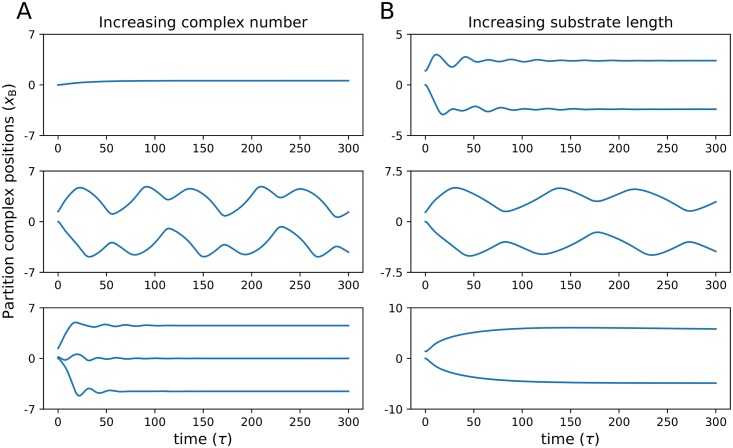
Partition complex dynamics have complex dependence on substrate length and complex number *in vivo*. (A) ParB-complex foci trajectories from simulations on a substrate of *l* = 14, *a*_tot_ = 0.17, *r* = 8, and *c* = 1 for increasing number of complexes from *N* = 1 (top), *N* = 2 (middle) and *N* = 3 (bottom). (B) ParB-complex trajectories from simulations of two foci on a substrate of increasing length while all other parameters are kept fixed at *a*_tot_ = 0.17, *r* = 8, and *c* = 1.0. The length of the substrate is increased from *l* = 10 (top) to *l* = 15 (middle) and then to *l* = 20 (bottom).

The counteracting effects on partition complex dynamics are spatial confinement and the amount of cytoplasmic ParA. For the constant rebinding case, the effects of partition complex number and system size on dynamics can be understood through a single mechanism—spatial confinement. This confinement, caused by increasing the number of complexes, the scale, *c*, or by decreasing the substrate length, subdues oscillatory behaviour. With variable ParA rebinding, an increase in complex number can lead to increased confinement that subdues oscillations but can also increase ParA availability, which encourages oscillatory motion. This makes it possible at a fixed amount of *a*_tot_ to observe a transition from a stationary (non-oscillatory) ParB-complex arrangement to oscillatory motion to back to stable fixed points as partition complex numbers increase. In Fig 6A we show this in simulations initiated with the same total ParA concentration and length (*a*_tot_ = 0.17, *l* = 10, *c* = 1, *r* = 8), changing only the complex number from one to three. We observe a transition from a decaying trajectory to oscillatory behaviour as partition complex number is doubled followed by a transition back into stable organization for three complexes. Similarly, an increase in substrate length may trigger oscillations, due to relaxation of confinement, or lead back to stable fixed points as free ParA is diluted out as the system size continues to increase. In Fig 6B we show results for a system of two complexes as only the length of the substrate is varied from *l* = 10 to *l* = 20 (*a*_tot_ = 0.16, *c* = 1.0, *r* = 8). The pair of complexes shows stable organization at *l* = 10, oscillates on a substrate length of *l* = 15, and returns to a stable arrangement at *l* = 20. Hence, the interplay of confinement effects and increased ParA availability creates counteracting effects that affect partition complex organization.

To put the above findings in context, total amounts of ParA in bacteria range from 100-4000 dimers which corresponds to *a*_tot_ = 0.05 to 2.3 in our model. With respect to length, nucleoid lengths vary from 800nm to 4*μ*m in WT *E. coli* cells corresponding to *l* = 8 − 40. These values reside near the boundaries shown in the above plots, and so these transitions in dynamics from oscillations to fixed points could be relevant *in vivo*.

### Diverse Par protein patterns in two-dimensions

So far we have only considered partition complex motion along a single dimension. Although the key dependencies of partition complex dynamics on system parameters remains the same, extending Eqs 3 and 4 to a two dimensional system (see supporting information for details) allows for a wider range of patterns to develop. We consider a limited concentration of ParA with a variable rate of rebinding (as *in vivo*) and include the experimentally observed anisotropic elasticity of the nucleoid in our model [24]. For a comprehensive portrait of possible patterns we change the amount of available ParA and alter the spatial confinement of the system by varying the substrate length and width. We first study the system under extreme confinement, then relax the confinement along one direction and eventually relax confinement in both directions for two different levels of ParA availability. Lastly, the parameter *c* changes the size of the depletion zone (smaller *c* corresponds to smaller depletion zones) and this also has the effect of affecting the confinement of the partition complex. So we also reduce confinement by decreasing the parameter *c*.

**Fig 7:**
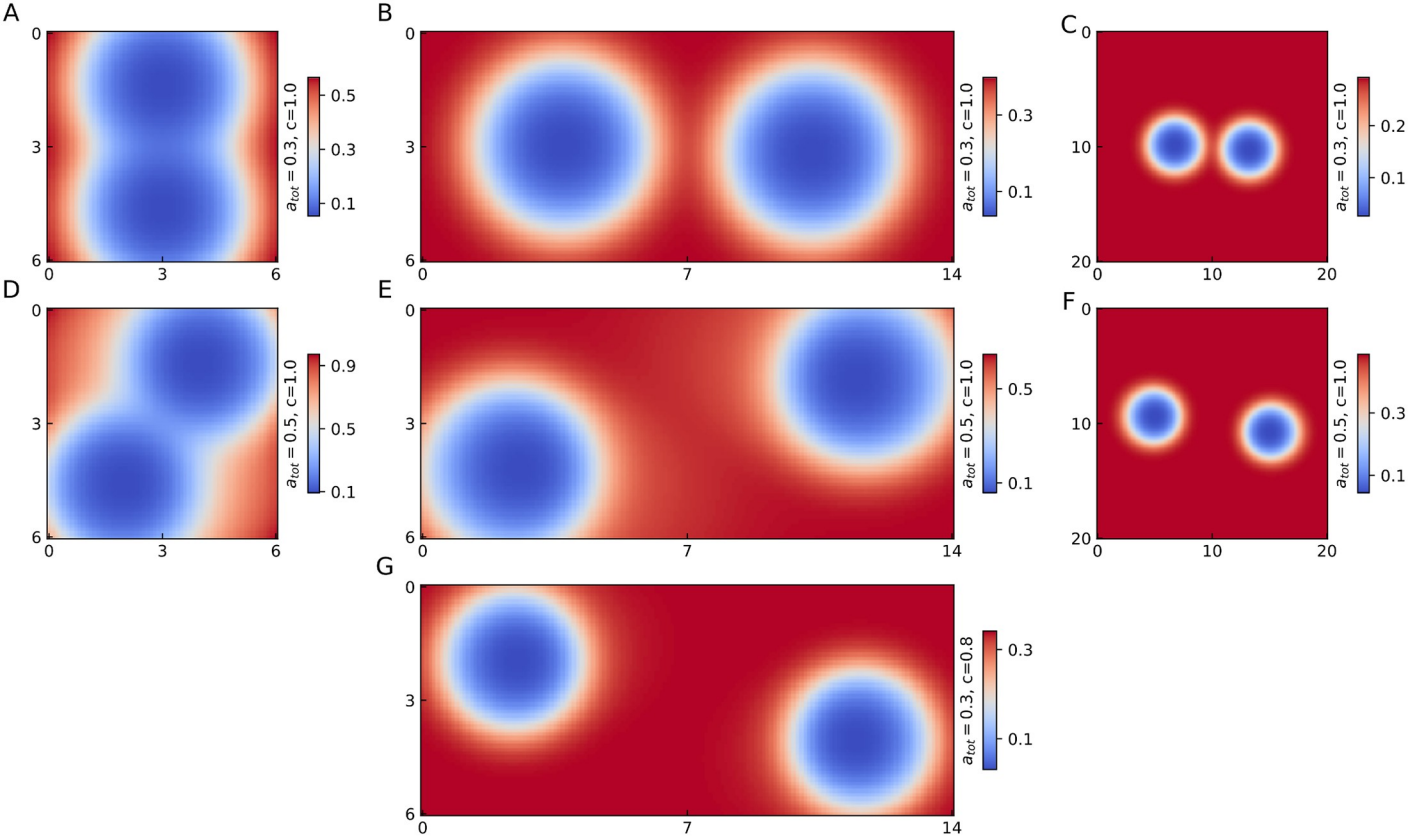
Organization patterns of two partition complexes on 2D substrates. (A) Bound ParA concentration for a simulated two partition complex system on a rectangular substrate of length 6 and width 6 with low ParA availability (*a*_tot_ = 0.3). (B) Same as (A) with length 14 and width 6. (C) Same as (B) with length = width = 20. (D) Same as (A) with higher ParA availability (*a*_tot_ = 0.5). (E) Same as (B) with *a*_tot_ = 0.5. (F) Same as (C) with *a*_tot_ = 0.5. (G) Same as (B) with *c* = 0.8. The partition complex trajectories along the x-axis and y-axis for these systems is given in S2 Fig.

Fig 7 shows the resultant ParA profiles for two identical partition complexes on two dimensional substrates of varying sizes. The instantaneous positional information of the partition complexes can be reliably inferred from the ParA depletion zones on each substrate (for trajectories of the centres of mass, see S2 Fig). Despite initializing the two partition complexes at similar locations on all substrates we observe a range of spatial patterns that depend on ParA availability and confinement of the partition complex. Fig 7A shows the final partition complex positioning on a substrate with a length of the order of the depletion zone of a single complex along both directions and low ParA concentration (*a*_tot_ = 0.3). Under this dual condition of extreme confinement and low ParA availability, the partition complexes align along the vertical axis due to the anisotropy in substrate elasticity (see supplementary information) and do not execute oscillations. As the substrate size is increased along the x-direction the partition complexes separate longitudinally, settling in stable positions at quarter length points due to a lack of ParA resources (Fig 7B). Finally, substrate size is increased along both directions and the partition complexes settle at a distance that minimizes any overlap in their respective depletion zones implying that oscillatory motion is prohibited by the lack of ParA resources (Fig 7C). On increasing the available ParA (*a*_tot_ = 0.5), the same system executes robust oscillations on the small substrate of aspect ratio one (Fig 7D) and on the larger substrate where confinement along the x-direction is reduced (Fig 7E). Axial asymmetry along the x-direction occurs as the two partition complexes are repelled (due to the depletion of ParA) along the y-direction at the point of closest contact in their respective trajectories (S2 Fig). These oscillations are eventually subdued as the size of the substrate is increased further and the two partition complexes settle at a distance that minimizes any overlap in their respective depletion zones (Fig 7F). This is similar to our finding in the previous section wherein systems with medium-low ParA showed oscillatory behaviour at small lengths which were subdued as the substrate length increased.

Interestingly, a decrease in the value of *c* from 1.0 to 0.8 triggers oscillations at low ParA (*a*_tot_ = 0.3) as shown in Fig 7G. Such a triggering of oscillations was not observed when the size of the system was increased for the same level of ParA (Fig 7C). Hence, modulating the value of *c* affects the partition complex environment in a complex manner and not just through a relaxation of confinement.

Recently, super-high resolution images have shown detectable differences between the spatial organization of a pair of F-plasmids in *E. coli* and the origins of replication of duplicate chromosomes in *B. subtilis* [18]. For both species, the respective partition complexes are under the control of the Par system and the nucleoids of the selected image samples were of equal length (∼1.5*μ*m). It was observed that the plasmid foci settle at quarter length positions along the length of the nucleoid and have axial symmetry, while the replicated chromosome origins in *B. subtilis* appear to segregate in the radial dimension and were also detected in diametrically opposite ends of the nucleoid farther than the quarter length points (see Fig 1 of [18]).

Considering a rectangular substrate as an approximation of a cylindrical nucleoid we have simulated the analogous substrate sizes (length = 1.4 *μ*m and width = 0.6 *μ*m) above for a two partition complex system (Fig 7B, 7E and 7G). We find remarkable similarity between the experimentally observed partition complex organization and the two possible bound ParA patterns for this substrate size. Low levels of ParA protein lead to stable positioning of partition complexes at the quarter length points while higher levels of ParA or a decrease in the value of the parameter *c* triggers oscillatory behaviour. We find that oscillatory motion leads to axial asymmetry, while stably positioned partition complex align symmetrically along the long axis for a substrate of this particular size. Oscillations lead to an asymmetry along the long axis due to inter-complex repulsion at the point of closest contact which reinforces segregation along the short axis and also pushes the partition complexes further towards the longitudinal extremes of the substrate (comparing B to E and G in S2 Fig). Hence, a difference in the ParA levels or the properties of the system that affect *c* could be the underlying cause behind the differences observed in *E. coli* plasmid foci and *B. subtilis* chromosomal origins pattern formation. A difference in the factor *c*, the ratio of the lengthscales of ParA removal and ParA-ParB force, can arise due to differences in the undetermined chemical rates at which ParA-ParB bonds are created and their decay. As previously mentioned, these rates are possibly affected by the structure of the partition complex, the ParB protein density on the complex, the interference of cytosolic ParA with the partition complex bound ParB or a combination of all these factors which may differ across bacterial species. It is important to note that while oscillatory dynamics of two partition complexes on rectangular substrates always creates axial asymmetry, it is possible to achieve stable positioning with axial asymmetry for shorter substrates corresponding to lengths much shorter (∼ 1*μ*m) than the size of the nucleoids considered in the experimental study.

## Discussion

In this paper we have mapped out the phase space of the dynamics of a deterministic model for ParB partition complexes on a finite sized substrate bound by ParA with applications to both, *in vivo* and *in vitro* systems. The resulting trajectories of the complexes could be classified into either oscillatory or decaying behaviour to stable equi-spaced positions along the substrate length, similar to what has been found in other simulation studies [19, 24, 25, 29]. Oscillations existed when the system parameters were such that sufficient ParA protein and substrate space were available to the ParB-complex. We found that when the available ParA was below a certain threshold, or if the rate of ParA hydrolysis was high, partition complex translocation was not triggered and the partition complexes moved to fixed positions due to their inter-complex repulsion. In addition to these factors substrate confinement due to multiple ParB complexes also affected the dynamics of partition complex foci in complex ways depending on ParA availability.

Using stability analysis on our coupled equations for ParA concentrations and ParB-complex positions, we provided a numerically calculated boundary separating oscillatory from non-oscillatory dynamics for a single ParB-complex for the extended parameter space including substrate length for a fixed *c*. These boundaries matched well with simulated results and assisted us in predicting system behaviour for a range of substrate lengths and ParA-ParB chemistry. Using the observation that ParB-complexes tended toward symmetric positions about the centre of the cell, we extended our one complex result to multiple complex cases. Our analytical approximation for the boundaries performed optimally for simulations in which complexes were spatially confined.

To mimic *in vivo* conditions, we considered the effect of limiting the available ParA such that the rebinding ParA concentrations were calculated for each simulation time step by subtracting the bound ParA from the total ParA, *a*_tot_, that the system was initialized with. This increased the effect of ParB stimulated hydrolysis rates on the available ParA, which in turn affected ParB-complex dynamics in complex ways. An increase in the number of complexes lead to counteracting effects of increased cytoplasmic ParA, which encouraged oscillations, or spatial confinement, which subdued oscillations. Similarly, an increase in substrate length could both, trigger oscillations due to relaxation of confinement as well as subdue oscillations due to redistribution of ParA over larger regions. In the context of a replicating and dividing cell, plasmids need to be able to sense and measure the growing cell which would necessitate oscillations, however oscillatory behaviour can favour the invasion of domains that is harmful to faithful segregation of genetic material. We argue that either through confinement (continuing to add plasmids) or via dilution (from growth) that the final end point is to achieve a stable fixed point dynamics that provides equi-distant spacing. If cells are poised with a total ParA concentration that is near the boundary of oscillations, it could achieve the necessary oscillations and then tend toward fixed point dynamics over the course of a single cell cycle.

In the context of chromosomes, the ParABS system, in conjunction with condensin complexes and organization proteins, orchestrates the post-replication spatio-temporal behaviour of the chromosomal origins in various bacteria. Fluorescence microscopy has shown how the chromosomal loci associated with the ParB-parS complex are positioned at specific locations along the length of the nucleoid during the process of replication followed by segregation. These patterns range from polar localization of the origin in *Caulobacter crescentus* [9], *Vibrio cholerae I* [30], and *Myxococcus xanthus* [31] to sub-polar fluctuations in *Pseudomonas aeruginosa* [32] and *B. subtilis* [11]. A host of mechanisms have been explored to explain the underlying reasons behind this variety in organization. By extending our model to two dimensions, and selecting a substrate of size analogous to the bacterial nucleoid we were able to obtain partition complex organization patterns with remarkable similarity to recently observed images of F-plasmids in *E. coli* and chromosome origins in *B. subtilis* [18]. At low level of ParA proteins the two partition complexes segregated longitudinally and settled symmetrically in the quarter length positions along the length of the substrate, like the F-plasmids. An increase in in the level of ParA resources or a decrease in the value of *c* was able to create the axial asymmetry observed for the chromosomal origins but it was accompanied by robust oscillations. Remarkably, the longitudinal oscillations of the *B. subtilis* chromosomes have been experimentally observed in another study and the role of the *par* loci in orchestrating these oscillations between *ori-ter* and left-*ori*-right states has been questioned [33]. Our study highlights the extent to which the Par system alone could be sufficient to explain the observed differences between plasmid and chromosomal organization in the specific case of *E. coli* and *B. subtilis* cells. In conclusion, the Par protein system is a versatile segregation machinery which is capable of organizing partition complexes in a variety of patterns based on the five system parameters considered in our model.

## Materials and methods

### Deterministic model of Par dynamics

For a complete description of the model, see the main text. The dimensionless equations, Eqs 3 and 4 were simulated to analyze the dynamics of the substrate bound ParA concentrations and the centre of mass of ParB bound cargo. In all simulations we used Δ*x* = 0.1 and Δ*τ* = 0.01. The ParA concentration on the substrate was initialized with an average initial concentration (*a*_tot_/2) and uniform noise with magnitude *δa* = 0.01 was added to each site. The resultant profile was a spatially noisy distribution about the mean ParA concentration. The ParB-complexes’ centre of mass coordinates, $x_{B}^{i}$, were chosen to be at random positions along the substrate length (-*l*/2, *l*/2). For the case of a constant ParA rebinding rate, the rebinding rate (*a*_tot_ − 〈*a*_*b*_〉) = *a*_tot_/(1 + *r*), while that for the case of limiting ParA resources, 〈*a*_*b*_〉 was calculated at every time step using 〈*a*_*b*_〉 = 1/*l*∫*a*(*x*, *τ*).

We used the initial value solver for a coupled system of ODEs from SciPy to integrate the differential equations for the complex positions and the concentration of ParA on the substrate. The time periods of the resulting trajectories were calculated by using a python module called Peakutils that detects peaks in single valued periodic functions. The time points of these peaks were used to determine amplitudes of oscillations and trajectories were classified as oscillatory if three consecutive peak to peak amplitudes were within a tolerance value of 0.01. Trajectories were classified as decaying if there was a monotonic decrease in their peak to peak amplitude. The details of the analytic approximation for the boundary separating parameter regimes that lead to complex oscillation or decay are given as supporting information.

For the extension of the model to two-dimensions (see supporting information for equations), a rectangular substrate was initialized with an average initial concentration, *a*_tot_/2 and spatial noise was added. At each time step, the mean bound ParA was calculated and subtracted from the total ParA concentration, *a*_tot_, to determine the available cytoplasmic ParA for rebinding. The equations determining the motion along the axial dimension and longitudinal dimension were altered to resemble nucleoid anisotropy and are provided as supporting information.

## Supporting information

S1 Text (PDF)

S1 Fig (TIF)

S2 Fig (TIF)

S1 Codes and Datasets (ZIP)
