# Supplementary material for: DNA segregation under Par protein control
Source: PLoS One. 2019 Jul 18;14(7):e0218520. doi: 10.1371/journal.pone.0218520 (PMC6638844; doi:10.1371/journal.pone.0218520)
Supplement: S1 Text — (PDF) [file pone.0218520.s001.pdf]

# DNA segregation under Par protein control

Lavisha Jindal and Eldon Emberly*

Physics Department, Simon Fraser University, Burnaby, British Columbia, V5A 1S6, Canada

*eemberly@sfu.ca

## Supporting information

**S1 Text. Model details and dedimensionalization.** Here we provide a full summary of the dynamical model and the resulting dimensionless equations. The substrate bound ParA concentration($A(X)$) is affected by three mechanisms: rebinding of cytoplasmic ParA-ATP, removal due to ParB stimulation in regions close to the partition complex centres ($X_B^i$) and random dephosphorylation of ParA-ATP into ParA-ADP which is un-initiated by PCs. As discussed in the main text, the rate of rebinding is $k_{on}$, the rate of stimulated hydrolysis is $\nu$ and the rate of random hydrolysis is $\gamma$. Given our proposal that bound ParA is hydrolysed based on its proximity to the partition complex we obtain the following equation for bound ParA as a function of position along the substrate length:

$$\frac{\partial A(X,t)}{\partial t} = k_{on}(A_{\mathrm{tot}} - \langle A_b \rangle) - \gamma A(X,t) - \sum_{i=1}^{N} \nu \exp[-\frac{(X - X_B^i)^2}{2(\sigma_F c)^2}]A(X,t). \quad (1)$$

The rate of rebinding $k_{on}$ has been experimentally found to be equal to the ParB stimulated rate of hydrolysis, $\nu$, which is approximated to be $0.1\mathrm{s}^{-1}$ [1]. Dividing the above equation by $\nu$ gives us the hydrolysis factor ($r = \nu/\gamma$) and we rescale actual time by $\nu$ and obtain a dimensionless time variable $\tau = \nu t$. We also rescale our ParA concentration by a reference concentration, $A_0$, such that $A_m(X,t) = A_0 a_m(x,\tau)$ and $A_{\mathrm{tot}} = A_0 a_{\mathrm{tot}}$. This reference concentration will be calculated later when we rescale the equations for ParB-complex foci motion. Finally, we rescale all spatial variables by the effective range of ParA fluctuations , $\sigma_F$, such that $x = X/\sigma_F$ is a dimensionless spatial variable. This gives us the following dimensionless equation for bound ParA:

$$\frac{\partial a(x,\tau)}{\partial \tau} = (a_{\mathrm{tot}} - \langle a_b \rangle) - \frac{a(x,\tau)}{r} - \sum_{i=1}^{N} \exp[-\frac{(x - x_B^i)^2}{2c^2}](x,\tau). \quad (2)$$

For the *in vitro* approximation for $\langle a_b \rangle$, we consider the steady state bound ParA concentration, $a^*(x)$, by considering the equation: $0 = a_{\mathrm{tot}} - \langle a_b \rangle - a^*(x)/r$, in the absence of any partition complex or spatial noise. For such a case $\langle a_b \rangle = a^*(x)$, as the substrate bound ParA would not have any spatial variation. This finally gives $\langle a_b \rangle = r a_{\mathrm{tot}}/(r+1)$. Thus, the average bound ParA is a function of total initial ParA and hydrolysis factor and we simulate the following equation to determine substrate bound concentration of ParA for an *in vitro* setup:

$$\frac{\partial a(x,\tau)}{\partial \tau} = \frac{a_{\mathrm{tot}}}{1+r} - \frac{a(x,\tau)}{r} - \sum_{i=1}^{N} \exp[-\frac{(x - x_B^i)^2}{2c^2}]a(x,\tau). \quad (3)$$

Now we use the aforementioned scaling factors to alter our equation for the change in ParB-complex positions, $X_B^i$. The complexes are translocating due to an elastic restoring forces that are exerted by the DNA bound ParA associating with them within a spatial range given by the characteristic nucleoid fluctuation length, $\sigma_F$. When a ParB complex at $X_B^i$ comes in contact with ParA-ATP at a position $X$, the elastic cost of deforming the system is $E_{\mathrm{el,f}} = \frac{1}{2}\frac{k_{\mathrm{B}}T}{\sigma_F^2}(X - X_B^i)^2$ where $R$ gives the effective range over which the elastic force extends. Thus the probability of forming ParA-ParB contacts is proportional to $e^{-E_{el,f}/k_{\mathrm{B}}T}$ and the total force is given by summing over concentration of ParA at all $X$. Assuming the complexes are overdamped without an inertial velocity, we get the following equation for a single partition complex focus:

$$\xi \frac{dX_B^i}{dt} = \int^{\text{all volume}} dV \exp[-\frac{(X - X_B^i)^2}{2\sigma_F^2}] \frac{k_{\mathrm{B}}T}{\sigma_F^2}(X - X_B^i)A(X,t), \tag{4}$$

where $\xi$ is the drag on the ParB-complex. To reduce our dimensions from a three dimensional volume (as inside the nucleoid) to a one dimensional system, we integrate the ParA concentration over the volume to give an integral along just the long axis of the nucleoid volume and rescale that ParA concentration by $A_0$ to obtain the dimensionless parameter $a(x,\tau)$ and the geometric factor $Q$. This is done as follows:

$$\int dV A(X,t) = \int dX Q \sigma_F^2 A_0 a(x,\tau). \tag{5}$$

Finally, rescaling all the spatial and temporal variables gives us:

$$\frac{dx_B^i}{d\tau} = \frac{QA_0\sigma_F k_{\mathrm{B}}T}{\xi\nu} \int_{-l/2}^{l/2} dx \exp[-\frac{(x - x_B^i)^2}{2}](x - x_B^i)a(x,\tau). \tag{6}$$

Now we evaluate the geometric factor, $Q$ that results from integrating out the other dimensions, reducing the system to a one-dimensional system aligned with the long-axis of the cell. We start by assuming that the leading ParA concentration is spherically symmetric at a given radial distance $(x - x_B)$. The force along the x-direction at a given angular position, $\theta$ and $\phi$ is $(x - x_B)\cos(\theta)$. Integrating over the half-sphere gives for the geometric factor:

$$Q = \int_0^{2\pi} \int_0^{\frac{\pi}{2}} d\theta d\phi \sin(\theta)\cos(\theta). \tag{7}$$

This gives a geometric factor of $Q = \pi$. To simplify the dimensionless equation we set the following term (with $A_0$ as length$^{-3}$) :

$$\frac{\pi\sigma_F k_{\mathrm{B}}T}{\xi\nu} A_0 = 1. \tag{8}$$

Using the Stokes-Einstein relation ($D\xi = k_{\mathrm{B}}T$) reduces the above to :

$$A_0 = \frac{\nu}{D\sigma_F\pi}. \tag{9}$$

From references [1,2] we take D = $0.0003\mu\mathrm{m}^2/\mathrm{s}$ and $\sigma_F = 100\mathrm{nm}$ for the diffusion coefficient and average longitudinal nucleoid fluctuation range in an *E. coli* cell. Considering the rate of random ParA dissociation to be $0.1\mathrm{s}^{-1}$ we find $A_0$ =1760nM as the reference concentration. This gives a simple dimensionless equation for the centre of mass of partition complexes:

$$\frac{dx_B^i}{d\tau} = \int_{-l/2}^{l/2} dx \exp[-\frac{(x - x_B^i)^2}{2}](x - x_B^i)a(x,\tau). \tag{10}$$

**S2 Text. Linear stability analysis of single partition complex dynamics** Here we linearize the dynamical equations for a single ParB-complex for a system with a constant rate of ParA rebinding (we do not consider the case of limited resources here) to examine the crossover from damped dynamics to oscillations. We assume that a steady state solution to the coupled equations exists and we linearize Eq. 3 for a single complex about this solution. The steady state solutions to the given equations are $x_B^* = 0$ and $a^*(x) = a_{\text{tot}}/[(1+r)(1/r + \exp(-x^2/2c^2))]$. For $x_B$ we consider a small time dependent perturbation $x_B(\tau) = x_B^* + \delta x_B(\tau)$. Instead of considering a spatial perturbation to the steady state ParA concentration, we assume that at small enough perturbations, the shape of the steady state solution does not vary much, rather only its central position moves. Thus we consider the time dependent variation of the ParA solution for small perturbations to go as $a(x,\tau) = a_{\text{tot}}/[(1+r)(1/r + \exp(-(x - \delta x_A(\tau))^2/2c^2)]$. The two dynamical variables are now $\delta x_B(\tau)$ and $\delta x_A(\tau)$. Putting these into the above two equations, we get the following linearized equations,

$$\frac{d\delta x_B}{d\tau} = \left[\pi a_{\text{tot}} \int_{-l/2}^{l/2} dx \frac{e^{-x^2/2}(x^2-1)}{(1+r)(1/r + e^{-x^2/2c^2})}\right] \delta x_B - \left[\frac{\pi a_{\text{tot}}}{c^2} \int_{-l/2}^{l/2} dx \frac{e^{-x^2/2} x^2 e^{-x^2/2c^2}}{(1+r)(1/r + e^{-x^2/2c^2})^2}\right]$$

and by integrating over the region, we get the following equation for the perturbation in the position of the minimum for the $a(x)$ distribution,

$$\frac{d\delta x_A}{d\tau} = \left[\frac{1}{l} \int_{-l/2}^{l/2} dx (1/r + e^{-x^2/2c^2})\right] \delta x_B - \left[\frac{1}{l} \int_{-l/2}^{l/2} dx (1/r + e^{-x^2/2c^2})\right] \delta x_A.$$

Assuming solutions of the form $\delta x_B(\tau) = \delta x_B \exp(\lambda \tau)$ and $\delta x_A(\tau) = \delta x_A \exp(\lambda \tau)$ yields an eigenvalue problem. The eigenvalues, $\lambda$ predict the nature of the dynamics for small perturbations away from the steady state solution: $\Re\lambda < 0$ are decaying solutions and $\Re\lambda > 0$ may yield oscillations. Our stability analysis has limitations as it considers only Hopf-type perturbations and assumes that a steady state exists. While it gives a good estimate of where the system crosses over from decaying to oscillatory solutions for most cases, it breaks down in the limit of low confinement. The values from this analysis was used to overlay the boundaries between oscillatory phase space and fixed point solution phase space in Fig 3.

**S3 Text. Two dimensional model for plasmid and chromosome organization *in vivo*.**

Extending our model to 2 dimensions requires careful consideration of the equations determining the motion of the partition complex along the longitudinal and axial dimension as the underlying nucleoid was observed to have anisotropic dynamics. The average DNA loci fluctuations along the nucleoid width have been measured to be smaller compared to fluctuations along the long axis ($\sigma_{F,x} = 100$nm, $\sigma_{F,y} = 50$nm) [1]. Furthermore, the reference concentration used in our model, $A_0$, is dependent on system dimensions and would change accordingly. The probability of a plasmid at $(X_B, Y_B)$ forming a contact with ParA at $(X, Y)$ in 2d is given by:

$$P(bond) = e^{(-\frac{1}{2}k_x(X-X_B)^2/k_B t - \frac{1}{2}k_y(Y-Y_B)^2/k_B t)}. \quad (11)$$

Substituting $k_x = \frac{k_B t}{\sigma_{F,x}^2}$ transforms the above to:

$$P(bond) = e^{(-\frac{1}{2}(X-X_B)^2/\sigma_{F,x}^2 - \frac{1}{2}(Y-Y_B)^2/\sigma_{F,y}^2)}. \quad (12)$$

We have from [1] that $\sigma_{F,x} = 100$nm while $\sigma_{F,y} = 50$nm implying that the chromosome is stiffer along the axial dimensions. Substituting this into the expression for P(bond) gives:

$$P(bond) = e^{(-\frac{1}{2}(x-x_B)^2 - 4.\frac{1}{2}(y-y_B)^2)}, \quad (13)$$

where all the length scales have been normalized by $\sigma_{F,x}$. The force due to the ParA springs is given by $-\vec{k}.\vec{r}$. Hence the force along x and y will be given by:

$$F_x = -k_x(X - X_B), F_y = -k_y(Y - Y_B), \quad (14)$$

or in the relevant constants:

$$F_x = -\frac{k_B t}{\sigma_{F,x}^2}(X - X_B), F_y = -\frac{k_B t}{\sigma_{F,y}^2}(Y - Y_B). \quad (15)$$

Rescaling the length scales by $\sigma_{F,x}$, time scale by $\nu$ and concentrations by $A_0$ as for the 1d case we have:

$$\frac{dx_B}{dt} = \int dxdye^{-\frac{1}{2}(x-x_B)^2 - 4.\frac{1}{2}(y-y_B)^2}(x - x_B)a(x, y), \quad (16)$$

$$\frac{dy_B}{dt} = 4\int dxdye^{-\frac{1}{2}(x-x_B)^2 - 4.\frac{1}{2}(y-y_B)^2}(y - y_B)a(x, y). \quad (17)$$

Where all the constants have been pulled out of the integral and the reference concentration, $A_0 = \nu/QD$, is chosen such that the equations are simplified to the form above. Consequently the removal of ParA concentration at any point (x,y) is given by:

$$\frac{da(x, y, \tau)}{d\tau} = (a_{\text{tot}} - \langle a_b \rangle) - \frac{a(x, y)}{r} - e^{-\frac{(x-x_b)^2}{2c^2} - 4\frac{(y-y_b)^2}{2c^2}}a(x, y). \quad (18)$$

**S1 Fig Phase diagram showing time periods for an unconfined *in vitro* system of $l = 20$.** The phase diagram of an *in vitro* system of length 20 with a constant ParA being supplied from the buffer for a single partition complex($c = 1$). While the time periods have marginally increased in magnitude compared the Fig 3A, the combined values of $a_{\text{tot}}$ and $r$ over which oscillations are triggered remains the same. Once the partition complex system is unconfined, the local gradient required to trigger oscillatory motion does not depend on system size or partition complex population.

**S2 Fig Partition complex trajectories on 2D substrates.** (A) The $x$ and $y$ coordinates of the centres of mass of two partition complexes on a substrate of $l_x = 6$ and $l_y = 6$ with $a_{\text{tot}} = 0.3$. (B) Same as (A) with $l_x = 14$ and $l_y = 6$. (C) Same as (B) with $l_x = l_y = 20$ and $l_y = 6$. (D) Same as (A) with higher ParA availability ($a_{\text{tot}} = 0.5$). (E) Same as (B) with $a_{\text{tot}} = 0.5$. (F) Same as (C) with $a_{\text{tot}} = 0.5$. (G) Same as (B) with $c = 0.8$. The bound ParA protein distribution shown in Fig 7 is the final protein distribution at the last time point shown in these trajectories.

## References

1. Surovtsev IV, Campos M, Jacobs-Wagner C. DNA-relay mechanism is sufficient to explain ParA-dependent intracellular transport and patterning of single and multiple cargos. Proceedings of the National Academy of Sciences. 2016;113(46):E7268–E7276.

2. Ietswaart R, Szardenings F, Gerdes K, Howard M. Competing ParA structures space bacterial plasmids equally over the nucleoid. PLoS computational biology. 2014;10(12):e1004009.
