# Supplementary figures and images for: DNA segregation under Par protein control
Source: PLoS One. 2019 Jul 18;14(7):e0218520. doi: 10.1371/journal.pone.0218520 (PMC6638844; doi:10.1371/journal.pone.0218520)

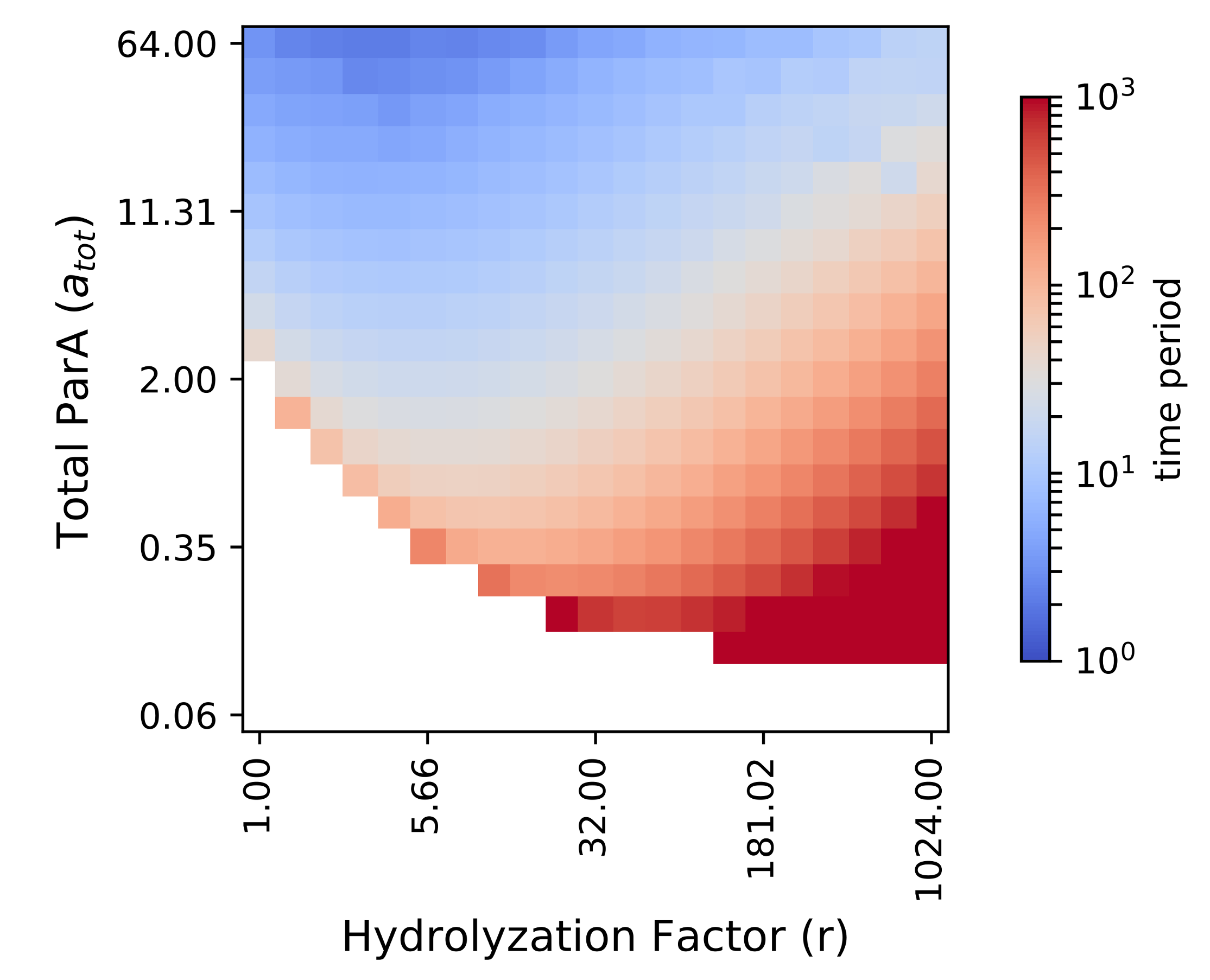

Supplement: S1 Fig — (TIF) [file pone.0218520.s002.tif]

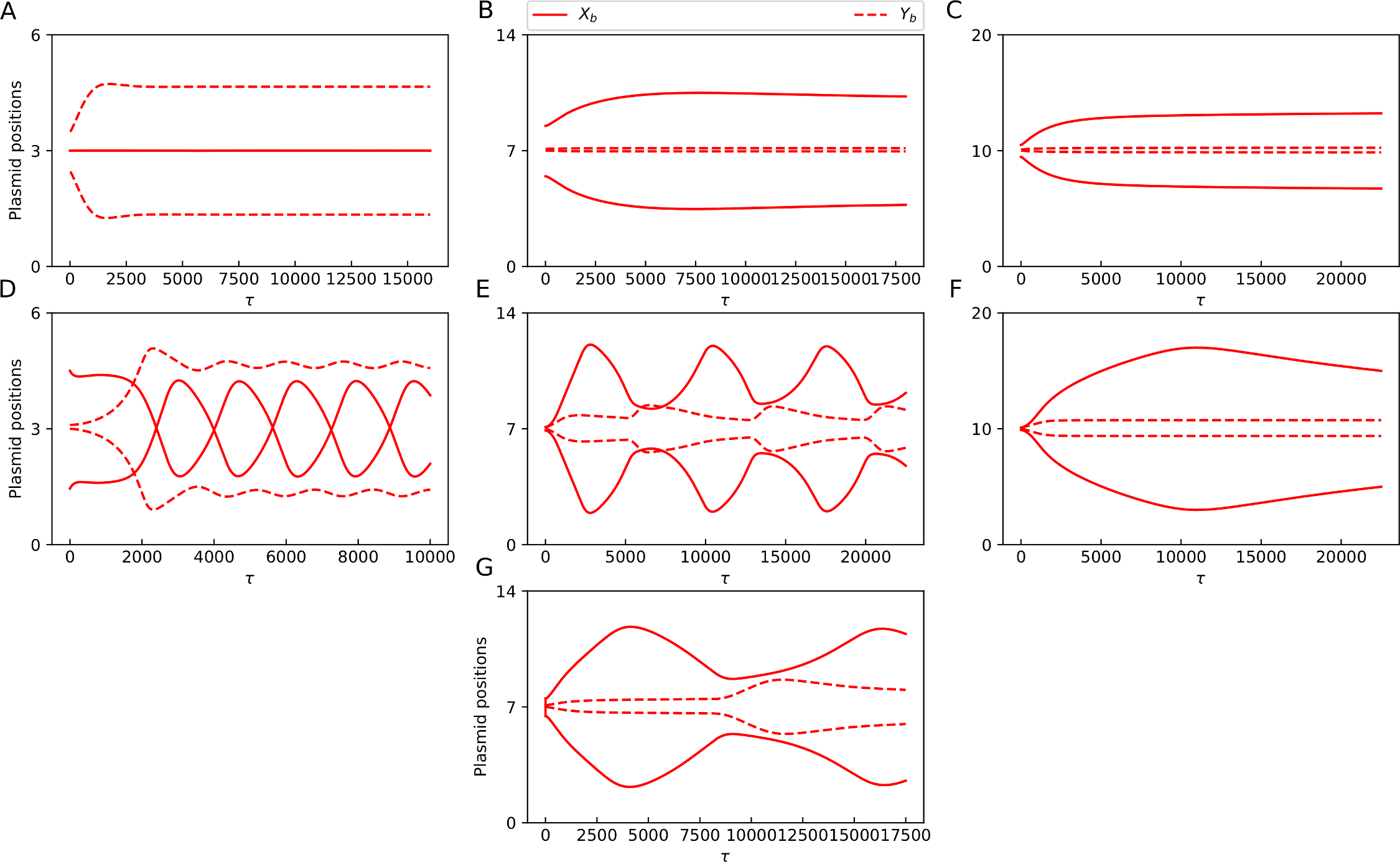

Supplement: S2 Fig — (TIF) [file pone.0218520.s003.tif]
